# The Landscape of SERCA2 in Cardiovascular Diseases: Expression Regulation, Therapeutic Applications, and Emerging Roles

**DOI:** 10.3390/biom16020247

**Published:** 2026-02-04

**Authors:** Jianmin Wu, Mengting Liao, Tengkun Dai, Guiyan Liu, Jiayi Zhang, Yiling Zhu, Lin Xu, Juanjuan Zhao

**Affiliations:** 1 Department of Immunology, Zunyi Medical University, Zunyi 563000, China; 2 Key Laboratory for Cancer Prevention and Treatment of Guizhou Province, Zunyi 563000, China; 3 Key Laboratory of Gene Detection and Treatment of Guizhou Province, Zunyi 563000, China

**Keywords:** cardiovascular disease, SERCA2, expression regulation, therapeutic targets

## Abstract

Driven by rapid socioeconomic progress and changing lifestyles, the global burden of cardiovascular diseases (CVDs) continues to escalate, with surging morbidity and mortality rates imposing a severe threat to public health. Clinical treatments are focused on the alleviation of treatments, highlighting the need for a deeper understanding of CVDs pathogenesis and the development of targeted therapies. Recent studies have identified imbalances in intracellular Ca^2+^ homeostasis as a key pathological mechanism in the progression of CVDs. Notably, sarcoplasmic/endoplasmic reticulum Ca^2+^-ATPase 2 (SERCA2), a membrane protein encoded by the *ATP2A2* gene and ranging from 97 to 115 kDa in molecular weight, plays a pivotal role in regulating intracellular Ca^2+^ levels. Extensive evidence links abnormal SERCA2 function to various CVDs, including heart failure, cardiac hypertrophy, atherosclerosis, and diabetic cardiomyopathy. This review systematically explores the regulatory mechanisms of SERCA2 expression and its functional regulation—including transcriptional regulation, post-translational modifications, and protein–protein interactions—and further investigates its pathological roles in cardiovascular diseases as well as its potential as a therapeutic target. By synthesizing current knowledge, this article aims to provide new insights for future basic research and establish a theoretical foundation for clinical applications.

## 1. Introduction

Cardiovascular disease (CVD) is the leading cause of death globally. According to the World Health Organization (WHO), approximately 18.6 million people died from CVD in 2023, accounting for 31% of all global deaths (WHO, 2024) [1]. Of these, ischemic heart disease (9.7 million cases) and stroke (6.1 million cases) are the leading causes of death. With the aging population and the prevalence of metabolic diseases such as diabetes and obesity, the annual number of CVD-related deaths is projected to exceed 23 million by 2030 [1]. Of particular concern is the fact that the CVD mortality rate in low- and middle-income countries is 2–3 times higher than that in high-income countries, highlighting the uneven distribution of healthcare resources. Economically, the global annual medical expenditure related to CVD has surpassed 1.49 trillion dollars [2], with approximately 60% allocated to the treatment of advanced complications [3]. This alarming situation underscores the urgent need to thoroughly analyze the molecular mechanisms underlying CVD and develop more effective early intervention strategies [4].

Recent studies have shown that an imbalance in intracellular Ca^2+^ homeostasis has been recognized as a common molecular feature of several CVDs [5]. Calcium ions, as important second messengers, are involved in the regulation of key physiological processes such as cardiac systolic and diastolic function, cell signaling, and apoptosis [6]. In cardiomyocytes, precise regulation of Ca^2+^ is essential for maintaining normal cardiac function. Sarcoplasmic reticulum/endoplasmic reticulum Ca^2+^-ATPase (SERCA) is the major intracellular calcium transport protein and plays a central role in maintaining calcium homeostasis. SERCA belongs to the P-type ATPases family, a class of membrane proteins that facilitate the transport of calcium ions into the lumen of the sarcoplasmic or endoplasmic reticulum, thereby maintaining low intracellular calcium levels during resting states [7]. In mammals, several SERCA isoforms exist, including SERCA1a-b, SERCA2a-c, and SERCA3a-f, encoded by the *ATP2A1*, *ATP2A2*, and *ATP2A3* genes, respectively [8]. These isoforms exhibit specific expression patterns across different tissues: SERCA2a is predominantly expressed in cardiomyocytes and type I skeletal muscle and is essential for cardiac contractile function, whereas SERCA1a is predominantly expressed in type II skeletal muscle [9]; SERCA3 is mainly expressed in non-muscle tissues such as pancreatic β-cells, endothelial cells, immune cells, and epithelial cells, where it contributes to the regulation of intracellular calcium homeostasis [10]. All SERCA proteins share a similar overall structure, comprising ten transmembrane (TM) helices (SERCA2b has eleven TM helices) and three cytoplasmic domains: the actuator (A) domain, the nucleotide-binding (N) domain, and the phosphorylated (P) domain [11]. Members of the SERCA family typically possess two Ca^2+^ binding sites formed by residues in TM4, TM5, TM6, and TM8 [12]. Unlike other isoenzymes, SERCA2b contains an additional TM helix and a subsequent lumenal extension tail, whose structural and functional roles have only recently begun to be elucidated [13]. Notably, SERCA2a shares 84% sequence homology with SERCA1a, suggesting that their Ca^2+^ transport mechanisms are highly conserved [14].

Among the various calcium-regulated proteins, SERCA2 is a key target due to its unique biological properties: (1) Molecular functionality: Each molecule of ATP hydrolyzed by SERCA2 translocates two Ca^2+^ ions against their concentration gradient into the sarcoplasmic reticulum, accounting for more than 70% of Ca^2+^ clearance in cardiomyocytes [8]; (2) Subtype specificity: SERCA2a, the dominant isoform in the myocardium, is tightly regulated by phospholamban (PLN), while SERCA2b, the dominant isoform in vascular tissues, exhibits a higher Ca^2+^ affinity but a lower turnover rate [15]. (3) Pathological implications: For example, Meyer and colleagues demonstrated that myocardial SERCA2a protein expression was reduced by 30–40% in heart failure patients, accompanied by a approximately 50% decrease in mRNA levels in cases of dilated cardiomyopathy [16]. In atherosclerotic plaques, SERCA2b dysfunction leads to increased endoplasmic reticulum stress in endothelial cells [17]. Additionally, the expression of a SERCA2a transgene has been shown to reverse cardiac dysfunction in animal models of heart failure [18]. SERCA2 oxidation at Cys674 is an early molecular event in ischemia–reperfusion injury [19]. Furthermore, SERCA2 forms nanoscale functional microdomains with the bridging-interconnecting protein BIN1, and disruption of this interaction contributes to the progression of heart failure [20].

In the cardiovascular system, SERCA2 facilitates cardiomyocyte relaxation and ensures the heart’s effective pumping function by transporting Ca^2+^ from the cytoplasm into the sarcoplasmic reticulum. The proper functioning of SERCA2 is essential for maintaining both cardiomyocyte contraction and relaxation [14]. Its expression and activity are regulated by a variety of factors, including hormones, neurotransmitters, and intracellular signaling pathways. A substantial body of evidence indicates that SERCA2 dysfunction is closely associated with a variety of cardiovascular diseases, such as heart failure, hypertension, and coronary heart disease, etc. Decreased expression or activity of SERCA2 leads to the accumulation of Ca^2+^ in cardiomyocytes, resulting in impaired cardiac contractile function.

**Figure 1:**
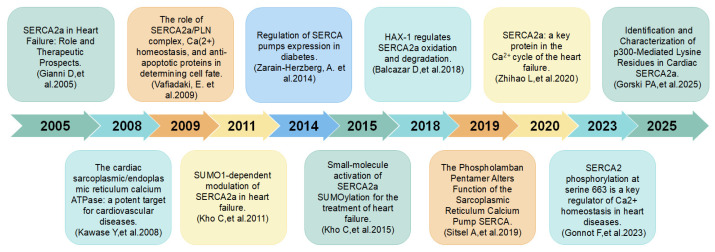
Overview of the Major Advances in Research on the Association Between SERCA2 and Cardiovascular Diseases [9,21,22,23,24,25,26,27,28,29,30].

Thus, SERCA2, as a member of the SERCA family and a key calcium ion pump, plays an important role in maintaining intracellular calcium homeostasis and regulating cardiomyocyte function. A comprehensive study of SERCA2 will not only enhance our understanding of the pathogenesis of cardiovascular diseases but also aid in the development of novel therapeutic strategies to improve cardiovascular health. Although significant progress has been made in the study of SERCA2 and cardiovascular disease, many key questions remain to be elucidated. Future studies need to further clarify the regulatory mechanisms governing SERCA2 expression and its specific roles in different cardiovascular diseases, aiming to provide new approaches for the prevention and treatment of these conditions. In this review, we systematically examine the latest advancements in SERCA2 research related to CVD, detail its expression regulation mechanisms and importance in pathophysiological processes, and explore new therapeutic strategies targeting SERCA2, thereby providing a theoretical foundation for overcoming current challenges in CVD treatment (Figure 1).

## 2. Summary of SERCA2

SERCA2, a crucial member of the sarcoplasmic reticulum/endoplasmic reticulum Ca^2+^-ATPase family, plays a pivotal role in the calcium cycle of cardiomyocytes. It is primarily responsible for actively transporting intracellular Ca^2+^ into the lumen of the sarcoplasmic or endoplasmic reticulum, thereby maintaining intracellular calcium homeostasis. This process is essential for various physiological functions, including cell signaling, energy metabolism, and muscle contraction and relaxation.

The SERCA2 protein is encoded by the *ATP2A2* gene, located on human chromosome 12q23-q24.1 [31], which comprises 22 exons. Alternative splicing of the *ATP2A2* gene generates different isoforms of SERCA2, including SERCA2a, SERCA2b, and SERCA2c, each exhibiting distinct expression patterns and functional roles across various tissues [32]. SERCA2a: Predominantly expressed in cardiac and skeletal muscle, SERCA2a is a core regulator of the cardiac calcium cycle. It is responsible for pumping calcium ions back to the sarcoplasmic reticulum, thereby terminating muscle contraction and facilitating diastole. SERCA2a accounts for over 70% of calcium transport activity in cardiomyocytes. SERCA2b: Widely distributed in non-muscle tissues such as the liver, pancreas, neurons, and endothelial cells, SERCA2b possesses a high affinity for calcium ions. It is primarily involved in maintaining endoplasmic reticulum calcium stores and regulating calcium signaling pathways [33]. SERCA2c: Expressed at lower levels, SERCA2c is suggested to play a compensatory role in conditions where SERCA2a or SERCA2b are deficient, particularly under pathological states [34].

The physiological function of SERCA2 is dependent on the hydrolysis of ATP, the energy of which is used to drive the transmembrane transport of calcium ions [35]. By lowering the concentration of calcium ions in the cytoplasm and replenishing calcium stores in the sarcoplasmic reticulum or endoplasmic reticulum, SERCA2 provides important support for processes such as cell signaling, energy metabolism, and muscle contraction. In particular, in the heart, SERCA2a takes up Ca^2+^ and returns it to the sarcoplasmic reticulum after each contractile maneuver completed by cardiomyocytes [36]. This process involves the realization of a typical “E1-E2 conformational cycle”: the E1 structure of SERCA2a has a high affinity for Ca^2+^ and its binding site (Asp351/Asp703) is exposed to the sarcoplasmic reticulum. However, the E2 structure has a lower affinity for Ca^2+^ and is located in the lumen of the sarcoplasmic reticulum. After cardiomyocyte diastole, Ca^2+^ is released from troponin into the cytoplasm and binds to E1 (the binding site is located at an aspartic acid residue), followed by phosphorylation of E1 with the involvement of ATP (Ca^2+^-E1-P), which results in a conformational change from E1 to E2 (Ca^2+^-E2-P) [37], and the E2 structure becomes an ADP-insensitive intermediate state [38]. This process allows the binding site for Ca^2+^ to be localized in the sarcoplasmic reticulum. Due to the low affinity of E2 for Ca^2+^, Ca^2+^ is released into the sarcoplasmic reticulum upon dissociation from E2, accompanied by the conversion of E2 to E1 and on to the next cycle [21]. The proper functioning of its function is essential for the systolic and diastolic cycles of the heart.

SERCA2 expression and activity are controlled by a sophisticated regulatory network at multiple levels. At the transcriptional level, transcription factors such as Sp1 [39] and NF-κB [40] directly bind to the promoter region of the *ATP2A2* gene, whereas the mitochondrial transcription factors TFAM and TFB2M indirectly regulate its expression through mitochondria-nucleus crosstalk [41]. In terms of epigenetic regulation, inhibition of histone deacetylase (HDAC) significantly upregulates SERCA2a expression. More importantly, multiple post-translational modifications dynamically regulate SERCA2 activity: including functional inactivation due to Cys674 oxidation, PLN phosphorylation deregulation effects, and aberrant nitroxylation modifications in pathological states. Notably, Blackwell et al. [42] further demonstrated that SERCA2a can form inherent homodimers in living cells, and crucially, the formation of such homodimers is not regulated by the conformational states of SERCA2a, PLN binding, or PLN phosphorylation. These regulatory mechanisms not only maintain the function of SERCA2 in the normal physiological state, but are also significantly altered during pathological processes such as diabetic cardiomyopathy and heart failure. Notably, cardiovascular risk factors such as oxidative stress, inflammatory response, and metabolic disorders can interfere with SERCA2 function through these regulatory nodes, forming a vicious circle and accelerating the progression of cardiovascular disease. This multilevel regulatory network not only ensures precise control of calcium homeostasis in cardiomyocytes, but also provides multiple potential targets for targeted intervention.

SERCA2 dysfunction plays a key role in the pathogenesis of several cardiovascular diseases. In patients with heart failure, SERCA2a expression is significantly reduced by 30–40%, leading to decreased efficiency of sarcoplasmic reticulum Ca^2+^ reuptake. This reduction is evidenced by a 2-3-fold prolongation of τ values, which in turn triggers intracellular calcium accumulation and results in both systolic and diastolic dysfunction in cardiomyocytes [43]. The development of atherosclerosis is closely associated with endothelial SERCA2b deficiency. This deficiency accelerates lesion progression by inducing a positive feedback loop between endoplasmic reticulum (ER) stress and inflammation [44]. In metabolic diseases such as diabetic cardiomyopathy, the hyperglycemic environment specifically inhibits SERCA2 activity through O-GlcNAc glycosylation modification [45]. Additionally, in the pathological process of pulmonary hypertension, abnormal function of vascular smooth muscle SERCA2 leads to calcium overload, which promotes pathological smooth muscle proliferation [46]. These findings confirm that abnormal regulation of SERCA2 is a common pathological basis for various cardiovascular diseases, including hypertension, cardiac hypertrophy, and coronary artery disease. Moreover, they suggest the therapeutic potential of targeted interventions aimed at specific SERCA2 dysfunction mechanisms in different diseases.

In summary, as a core molecule regulating intracellular calcium homeostasis, abnormal function of SERCA2 is closely related to the pathogenesis of various cardiovascular diseases such as heart failure, atherosclerosis, diabetic cardiomyopathy, and pulmonary hypertension. Existing studies have shown that decreased expression, reduced activity or post-translational modification of SERCA2 can lead to an imbalance of intracellular calcium homeostasis. This imbalance subsequently triggers myocardial contractile dysfunction, vascular endothelial injury, and abnormal smooth muscle proliferation. Elucidating the regulatory mechanisms of SERCA2 in cardiovascular diseases not only helps reveal the molecular basis of disease development but also provides a crucial theoretical foundation for developing novel therapeutic strategies targeting SERCA2 (Table 1).

In this review, we systematically examine the research progress on SERCA2 in cardiovascular diseases, spanning from molecular mechanisms to clinical translation. We focus on its expression regulatory network, pathophysiological roles, and therapeutic potential, and propose future research directions aimed at promoting precision prevention and treatment of cardiovascular diseases.

## 3. The Regulatory Mechanisms of SERCA2 Expression and Its Functional Regulation

**Figure 2:**
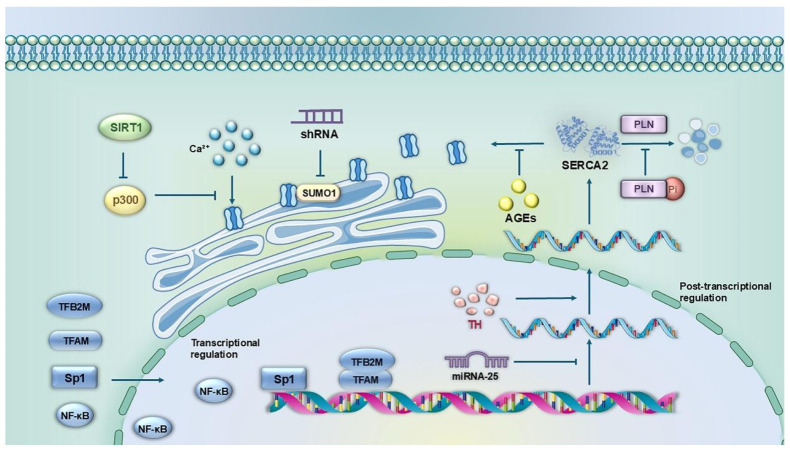
The regulatory mechanisms of SERCA2 expression. The regulatory mechanism of SERCA2 expression is divided into two main parts: transcriptional regulation and post-transcriptional regulation. The mitochondrial transcription factors TFB2M and TFAM are mainly involved in transcriptional regulation. Thyroid hormones and MicroRNAs can affect SERCA2 mRNA expression. Phosphorylation, SUMO modification plays a role in post-transcriptional regulation. shRNA can inhibit SUMO1. AGEs can inhibit the activity of SERCA2a. More details were shown in the text.

The regulation of SERCA2 expression constitutes a complex network that operates at multiple levels, including transcriptional control, post-transcriptional modifications, and protein stability regulation. These regulatory mechanisms jointly maintain the balance of intracellular calcium homeostasis. Disruptions in this intricate balance are closely associated with the pathogenesis of various cardiovascular diseases. An in-depth analysis of the regulatory mechanisms governing SERCA2 expression is crucial not only for elucidating the molecular basis of cardiovascular disease development but also for providing a theoretical foundation for the creation of targeted therapeutic strategies (Figure 2).

### 3.1. Transcriptional Level Regulation

Transcriptional regulation of SERCA2 is mainly dependent on the interaction of specific transcription factors with the promoter region of the *ATP2A2* gene. Studies have shown that mitochondrial transcription factor A (TFAM), and mitochondrial transcription factor B2 (TFB2M) are key transcription factors regulating *ATP2A2* gene expression in cardiomyocytes [47]. In terms of molecular mechanisms, TFAM and TFB2M specifically bind to the *ATP2A2* promoter regions −122 bp to −114 bp and −122 bp to −117 bp, respectively [47]. Functional studies showed that overexpression of TFAM and TFB2M increased the transcriptional activity of SERCA2a by approximately 2-fold and significantly attenuated the stress-induced reduction in SERCA2a mRNA levels, suggesting a protective role in maintaining the homeostasis of SERCA2a expression. Notably, TFAM expression was significantly downregulated in pathological states such as diabetic cardiomyopathy, myocardial infarction, and heart failure, which directly led to the inhibition of SERCA2a transcription and became one of the important molecular mechanisms of SERCA2 dysfunction.

In addition, the transcription factor Sp1 has also been shown to be involved in the transcriptional regulation of the *ATP2A2* gene. Sp1 mainly acts in the proximal promoter region (−284 bp to −80 bp) of the *ATP2A2* gene, and Sp1-mediated transcriptional regulation may be a key factor contributing to the decrease in the level of SERCA2a mRNA under pathological conditions such as pressure overload [39]. Particularly in diabetic cardiomyopathy, abnormalities in the regulatory pathway are likely to be important for the downregulation of SERCA2a expression, although the precise molecular mechanisms remain to be further elucidated [39].

### 3.2. Regulatory Mechanisms at the Post-Transcriptional Level

The post-transcriptional regulation of SERCA2 is a delicate and complex process, mainly involving mRNA splicing, stability regulation and translation efficiency regulation. Recent studies have shown that MicroRNAs play a key role in the post-transcriptional regulation of SERCA2a expression. These non-coding RNA molecules of approximately 22 nucleotides in length specifically bind to the 3′ untranslated region (3′-UTR) of SERCA2a mRNA through their seed sequences, which in turn regulate the stability and translation efficiency of the mRNA [48]. Experimental evidence has shown that adenovirus-mediated overexpression of MiRNA-25 (50% increase) resulted in a significant 35% reduction in SERCA2a protein levels, accompanied by progressive deterioration of cardiac contractile function [49]. In contrast, inhibition of endogenous MiRNA-25 by antisense oligonucleotides resulted in a 50% increase in SERCA2a protein expression [49]. In addition, Gurha and colleagues [50] found that MiRNA-22 knockout mice exhibited significant calcium handling dysfunction, including a prolonged cytoplasmic Ca^2+^ decay time and a 25% reduction in sarcoplasmic reticulum Ca^2+^ loading, compared with wild-type mice. These findings highlight the important role of MiRNAs in the regulation of SERCA2a expression, although the precise mechanism of action remains to be further elucidated.

In terms of mRNA stability regulation, several studies have confirmed the significant positive regulation of SERCA2a expression by thyroid hormones [51]. Thyroid hormone treatment increased SERCA2a mRNA levels by 80% to 167% in rodent and rabbit cardiomyocytes [52], whereas myocardial SERCA2a mRNA levels were reduced by 28% to 64% in hypothyroid animal models [53]. This regulation was equally significant at the protein level, consistent with changes in mRNA, from an 88% to 134% increase in myocardial SERCA2a protein expression in hyperthyroid rats [54], compared to a 74% decrease in hypothyroid rats [55]. Molecular mechanistic studies have shown that this regulation is mainly achieved through transcriptional activation mediated by three independent thyroid hormone response elements [56].

In addition, the adipocyte-derived peptide hormone lipocalin has been shown to modulate SERCA2a function through multiple mechanisms. Guo and colleagues [57] showed that globular lipocalin treatment increased the level of phosphorylated PLN protein by approximately 5-fold in rat left ventricular tissues, thereby rescuing the inhibitory effect of PLN on SERCA2a. The cardioprotective effect of lipocalin may be achieved in part through the AMPK signaling pathway [58], which has been shown to regulate SERCA1a protein and SERCA2a mRNA levels in mouse skeletal muscle [59]. These findings provide a new perspective for understanding the post-transcriptional regulatory network of SERCA2a and a theoretical basis for developing relevant therapeutic strategies.

### 3.3. Mechanisms of Post-Translational Modification Regulation at the Protein Level

At the protein level, the activity of SERCA2 is precisely regulated by a variety of post-translational modifications (PTMs), which are covalent chemical modifications that occur after protein synthesis, significantly expanding the structural and functional diversity of proteins by adding functional groups (e.g., phosphoryl groups, acetyl groups, etc.) to specific amino acid residues [60]. More than 400 PTMs have been identified in eukaryotes, and these modifications can occur at different sites of the same protein, forming a complex “modification code” that precisely regulates protein activity, stability, subcellular localization and interaction networks [61,62].

Glycosylation modifications play an important role in the regulatory network of PTMs of SERCA2a. Glycosylation, as an important post-translational modification of proteins, usually occurs at specific locations in the endoplasmic reticulum and Golgi apparatus. It is mainly divided into two types: N-glycosylation and O-glycosylation. Compared with other post-translational modifications, glycosylation has unique biological significance, including complex structural diversity, and evolutionary conservation. Glycosylation modifications have important implications for protein stability, cell adhesion and recognition, intracellular signaling, and epigenetics, thus participating in the regulation of cell biology and pathogenesis [63]. Glycosylation has been shown to reduce SERCA2a activity, thereby decreasing Ca^2+^ reuptake after myocardial contraction. Bidasee and colleagues [64] found that advanced glycosylation end-products (AGEs) produced under diabetic conditions can covalently bind to SERCA2a intracellularly, which, by disrupting its internal and interdomain tertiary structure, leads to a decrease in SERCA2a activity, significantly inhibiting Ca^2+^ transport activity. Molecular mechanistic studies indicated that AGEs modification interfered with the dynamic equilibrium of SERCA2a conformational changes and hindered its E1-E2 conformational transition process. Of particular note, O-GlcNAc glycosylation, as a dynamic and reversible modification, regulates SERCA2a function through two mechanisms: on the one hand, it directly modifies the SERCA2a protein and affects its stability; on the other hand, it indirectly alters the SERCA2a activity by regulating the phosphorylation state of PLN [65]. This dual regulatory mechanism is particularly important under metabolic stress conditions and provides a new molecular perspective for understanding diabetes-associated cardiac function abnormalities.

Oxidative stress-induced tyrosine nitration is also an important mechanism for regulating SERCA2a activity [66]. Li and colleagues [67] found that in a diabetic cardiomyopathy model, iron overload led to elevated levels of reactive oxygen species (ROS), which in turn induced a nitration modification of the tyrosine residues of SERCA2a. This modification impairs the calcium transport capacity of SERCA2a by decreasing its affinity for Ca^2+^. Notably, Lokuta et al. [68] found approximately 2-fold elevated nitrotyrosine levels in cardiac tissues of dilated cardiomyopathy (DCM) patients compared to controls and confirmed SERCA2a as the major nitration target protein by mass spectrometry, which may be related to long-term exposure to peroxynitrite. In addition, Lancel et al. [19] demonstrated that nitroxyl (HNO) activates SERCA in cardiomyocytes via glutathiolation of cysteine 674, which elevates the calcium transport efficiency of the enzyme and ultimately ameliorates the contractile and relaxant functions of cardiomyocytes. Meanwhile, Sivakumaran et al. [69] reported that HNO can also augment SERCA2a activity and cardiomyocyte function by facilitating redox-dependent oligomerization of PLN. In cardiomyocytes, HNO maintains PLN in an oligomeric conformation through disulfide bond-mediated mechanisms; this process decreases the level of free PLN monomers with inhibitory activity, thereby relieving the inhibitory effect of PLN on the calcium pump [70]. Furthermore, utilizing ^15^N-edited nuclear magnetic resonance (NMR) spectroscopy, Keceli et al. [71] identified that HNO exerts its cardioprotective effect by targeting cysteine 41 (Cys41) and cysteine 46 (Cys46) residues of PLN to improve cardiac function. Collectively, these redox-sensitive modifications underscore the sophisticated and intricate regulatory mechanisms governing SERCA2a activity.

In the cardiovascular system, sumoylated modification of SERCA2a is a highly regulated post-translational modification process [72] mediated by a conserved enzyme cascade reaction (E1-activating enzyme, E2-binding enzyme UBC9, and E3 ligase) [73], which covalently binds to specific lysine residues (K480, K571, and K585) via SUMOs (primarily SUMO1 isoforms), and reversibility is dynamically regulated by SENPs proteases [74].

Kho and colleagues [22] found that SUMO1 expression and sumoylation levels of SERCA2a were significantly downregulated in patients and mouse models of heart failure, and inhibition of SUMO1 by small hairpin RNA (shRNA) accelerated the deterioration of cardiac function. Molecular mechanistic studies have shown that SUMO1 mainly modifies the K480, K571, and K585 loci of SERCA2a [75], and this modification is disease stage-specific: it is mildly upregulated during compensated cardiac hypertrophy and drastically decreased during decompensation [76]. Kho and colleagues [77] further found that MiR-146a negatively regulated SUMO1 expression through inhibition of the SUMO1-SERCA2a axis. Notably, the small molecule compound N106 could promote SUMCA2a sumoylation through activation of E1 enzymes and significantly improve cardiac function [23], which provides a new strategy for targeting the sumoylation pathway for the treatment of heart failure. These findings not only elucidate the critical and disease-stage-specific role of sumoylation modification in maintaining SERCA2a function, but also provide potential therapeutic targets for the treatment of cardiovascular diseases [78].

Protein acetylation modification is an important post-translational modification mechanism that precisely regulates protein function by enzymatically or non-enzymatically transferring acetyl groups to specific amino acid residues of target proteins [79]. Depending on the modification site, acetylation modifications can be divided into two categories: N-terminal α-aminoacetylation (catalyzed by N-acetyltransferases NATs) and lysine ε-aminoacetylation (catalyzed by lysine acetyltransferases KATs) [80]. Among them, lysine acetylation, as a dynamic and reversible modification, is co-regulated by KATs and lysine deacetylases (KDACs), which play a central role in transcriptional regulation, metabolic reprogramming, and signal transduction [81].

In the cardiovascular system, the acetylation modification of SERCA2a is closely related to its functional regulation. Studies have shown [82] that in cases of heart failure, the expression of SIRT1 (a class III histone deacetylase) is reduced, leading to a significant increase in the acetylation level of SERCA2a, especially at lysine 492 (K492). Molecular mechanism studies have shown that p300-mediated K492 acetylation impairs the calcium transport activity of SERCA2a by decreasing its ATP-binding capacity [24]. Notably, SERCA2a acetylation exhibits antagonistic effects with sumoylation, and this inter-modification crosstalk provides a new target for heart failure therapy: on the one hand, SERCA2a acetylation can be reduced by inhibition of p300 activity; on the other hand, the deacetylation can be enhanced by activation of SIRT1, thus restoring SERCA2a function [24,82]. These findings not only reveal the pathological significance of protein acetylation modification in cardiovascular diseases, but also lay a theoretical foundation for the development of therapeutic strategies targeting acetylation regulation.

In addition to this, the functional regulation of SERCA2a involves a variety of important post-translational modifications and protein interactions. Quick and colleagues [83] identified for the first time, through proteomic analysis, that SERCA2a interacts with striated muscle preferentially expressed protein kinase (SPEG) and confirmed this regulatory mechanism in primary cardiomyocytes. Further research [84] has demonstrated that the second kinase domain of SPEG mediates the phosphorylation of SERCA2a at the Thr484 site. This post-translational modification promotes the assembly of SERCA2a into oligomers, which in turn enhances SR/ER Ca^2+^ reuptake efficiency in both cultured cells and primary neonatal mouse cardiomyocytes.

In addition to phosphorylation modification, the activity of SERCA2a is finely regulated by a variety of regulatory proteins. As a major inhibitor of SERCA2a, phosphorylation of PLN at Ser16 (PKA-mediated) and Thr17 (CaMKII-mediated) sites deregulates the inhibitory effect, increasing SERCA2a activity by 2-3-fold, accelerating the rate of diastole and contributing to the diastolic effect produced by high intramyocardial calcium ion concentrations and β-adrenergic stimulation [85]. Sarcolipin (SLN), a homolog of PLN, interacts with SERCA2a through a conserved transmembrane structural domain and reduces its apparent affinity for Ca^2+^. Serine/threonine kinase 16 (STK16)-mediated phosphorylation of the SLN Thr5 site promotes SLN-SERCA2a dissociation and enhances β-adrenergic-stimulated diastolic function [86]. Additionally, the Olson group [87] performed a bioinformatics screen of the mouse genome to identify open reading frames (ORFs) potentially containing SERCA-binding motifs homologous to those of myoregulin (MLN),P LN, and SLN. This screen led to the discovery of two genes encoding uncharacterized transmembrane micropeptides, both harboring SERCA-binding motifs, which were ultimately named endoregulin (ELN) and another regulator (ALN), respectively. Subsequently, the Olson group [88] further evaluated the regulatory effects of multiple micropeptides—including PLB, SLN, ELN, dwarf open reading frame (DWORF), MLN, and ALN—on SERCA. These analyses employed co-immunoprecipitation and fluorescence resonance energy transfer (FRET) to quantify micropeptide oligomerization and SERCA binding. Results showed that all micropeptides, except DWORF, reduced the apparent affinity of SERCA for Ca^2+^. Interestingly, DWORF exhibits unique oligomerization/SERCA-binding properties: unlike other SERCA-interacting micropeptides that exert inhibitory effects, DWORF does not suppress SERCA activity upon binding [89]; instead, it promotes Ca^2+^ uptake by competitively displacing PLB [90], which stands in stark contrast to the inhibitory functions of other SERCA-binding micropeptides.

The apparent Ca^2+^ affinity of cardiac SERCA2a can be further reduced by direct interaction with one or both of the two associated small TM (transmembrane) proteins, i.e., PLN in the atria and ventricles, and myocardial lipoproteins in the atria [85]. In recent years, it has been found that proteins such as HAX-1, HRC and S100A1 can also regulate the activity and stability of SERCA2a by interacting with it, and these findings further highlight the central role of SERCA2a in the myocardial calcium cycle. This multilevel regulatory network enables SERCA2a to respond precisely to various physiological and pathological stimuli and maintain normal cardiac function.

In summary, the regulation of SERCA2 expression constitutes a highly complex multilayered network, covering multiple levels of transcriptional regulation (e.g., the roles of transcription factors such as TFAM, Sp1, etc.), post-transcriptional modifications (including MiRNA-mediated regulation and mRNA stability control), and post-translational modifications of proteins (e.g., phosphorylation, sumoylation, and acetylation, etc.). Recent research advances have shown that the abnormal function of SERCA2 is not only closely related to traditional cardiovascular diseases such as heart failure and cardiac hypertrophy, but also plays a key role in pathological processes such as pulmonary hypertension, atherosclerosis and diabetic cardiomyopathy. These findings have important medical applications: (1) they provide a new theoretical framework for understanding the molecular mechanisms of cardiovascular diseases; (2) they reveal the potential value of SERCA2 as a therapeutic target; and (3) they point the way to the development of precision therapeutic strategies. It also suggests new research focuses for future studies: (1) crosstalk mechanism between different PTMs; (2) resolution of tissue-specific regulatory networks; and (3) clinical translation of novel therapeutic regimens based on SERCA2 regulation. The increasing findings not only provide new perspectives for our comprehensive understanding of the pathogenesis of cardiovascular diseases, but also lay a solid foundation for the development of targeted therapeutic strategies against SERCA2.

## 4. Role of SERCA2 in Cardiovascular Diseases

**Figure 3:**
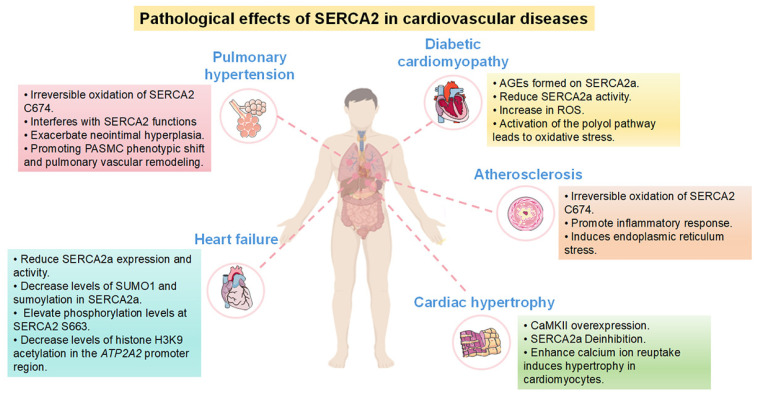
Pathological effects of SERCA2 in cardiovascular diseases.

SERCA2, as an important calcium ion pump, plays a key role in the cardiovascular system and is involved in both contraction and diastole of cardiomyocytes and is essential for the functional regulation of vascular smooth muscle. It regulates the contractility and diastolic capacity of the heart through a fine-grained calcium signaling network, thereby affecting overall cardiovascular health. Dysfunction of SERCA2 is closely associated with the development of a variety of cardiovascular diseases, including heart failure, pulmonary hypertension, and atherosclerosis. Therefore, an in-depth study of the specific mechanism of SERCA2’s role in cardiovascular disease is important for developing therapeutic strategies targeting SERCA2 and improving the prognosis of patients with cardiovascular disease (Figure 3).

### 4.1. SERCA2 and Pulmonary Arterial Hypertension

Pulmonary hypertension (PH) is a common cardiopulmonary vascular remodeling disorder characterized by progressive remodeling of small distal precapillary pulmonary arterioles and muscularization of peripheral arteries, leading to increased pulmonary vascular resistance, right ventricular hypertrophy and failure [91]. Studies have shown that SERCA2 plays an important role in the development and progression of PH. The transition of pulmonary artery smooth muscle cells (PASMC) from a contractile to a synthetic phenotype is key to pulmonary vascular remodeling, as evidenced by thickening of the vascular layer [92]. Elevated intracellular Ca^2+^ levels are considered to be one of the main factors promoting the proliferation of PASMCs. As an important node in the intracellular Ca^2+^ signaling pathway network, SERCA proteins play a central role in the cardiovascular system.

SERCA2, the predominant isoform in the cardiovascular system, contains multiple cysteines, with cysteine 674 (C674) on the cytoplasmic side of the cell being the major regulatory site for its activity. Unlike the common way of sulfhydryl existence, the sulfhydryl group of SERCA2 at the C674 position is surrounded by a pocket structure formed by positively charged arginine (R), resulting in the sulfhydryl group existing in an ionic state (-S-) [40], which is highly susceptible to ROS/RNS [93]. In PASMC, NADPH oxidase 4 (Nox4) promotes irreversible oxidation of cysteine (C674) at position 674 of SERCA2 through the generation of ROS, thereby exacerbating neointimal hyperplasia caused by vascular injury [94]. In addition, the irreversible oxidation of C674 (C674-SO3H) in SERCA2 exacerbates the formation of aortic aneurysm and atherosclerosis [95]. Tong and colleagues [96] found that the C674-SO3H in SERCA2 was significantly increased in hypoxia-induced PH, which resulted in the irreversible oxidation of C674, a key redox site, and interfered with the function of SERCA2. thereby promoting pulmonary vascular remodeling and the development of PH.

Interestingly, the irreversible oxidation of C674 did not affect the mRNA and protein expression levels of SERCA2, but upregulated cyclin (A1, A2, B1) and cyclin-dependent kinase (CDK) (cyclin-dependent kinase, CDK) pathways through the activation of the inhibitor of the inositol-requiring enzyme-1alpha (IRE1alpha) and spliced X-box-binding protein-1 (XBP1s) pathways. XBP1s pathway, upregulates cell cycle-related proteins such as cyclin (A1, A2, B1) and cyclin-dependent kinase (CDK) (CDK 1 and CDK 2), thereby accelerating the cell cycle and promoting cell proliferation. In addition, the activated calmodulin phosphatase (CaN)/nuclear factor of activated T-cells (NFAT) pathway contributes to the pathogenesis of wild lily alkaloids-induced pulmonary hypertension in rats [97]. In this study, it was found that dysfunction of SERCA2 activated the Ca^2+^-dependent CaN-mediated NFAT4 pathway, which directly led to PASMC phenotypic transition, whereas activation of SERCA2 by CDN1163 prevented PASMC phenotypic transition and pulmonary vascular remodeling in SKI mice. In PH-susceptible conditions such as hypoxia, the large amount of ROS generated irreversibly oxidizes C674, resulting in the irreversible oxidation of the redox site C674, which interferes with the function of SERCA2 and promotes pulmonary vascular remodeling and PH, whereas overexpression of SERCA2a S674 or SERCA2b S674 activates the IRE1α-XBP1s pathway to promote the proliferation of pulmonary arterial smooth muscle cells, thereby causing pulmonary vascular remodeling. thereby causing pulmonary vascular remodeling. In conclusion, it is suggested that SERCA2 dysfunction, especially SERCA2b, can also activate the cytoplasmic calcium-dependent CaN/NFAT4 pathway to promote PASMC phenotypic transition and pulmonary vascular remodeling. SERCA2 agonists have therapeutic potential for the prevention and treatment of pulmonary vascular remodeling and PH in the clinical setting.

### 4.2. SERCA2 and Heart Failure

Heart failure (HF) is the leading cause of hospitalization and death in developed countries and is defined as the inability of the heart to supply enough blood to meet the demands of the organism. Poor contractile performance of failing cardiomyocytes is usually associated with abnormal Ca^2+^ cycling, especially reduced Ca^2+^ content in the SR [25], and SERCA2 is a key regulator of intracellular Ca^2+^ concentration homeostasis. It has been reported that SERCA2a, the myocardial-specific variant of SERCA2, is a key determinant of cardiac function, and its expression is significantly down-regulated in myocardial tissues of patients with heart failure [22]. However, emerging evidence [98], highlights significant variability in SERCA2a expression in heart failure with preserved ejection fraction (HFpEF), which accounts for 50% or more of all HF cases. Unlike the consistent downregulation in HFrEF, SERCA2a protein and mRNA levels in HFpEF patients are not uniformly decreased—some studies report no significant change compared to healthy controls, while a subset show mild upregulation or focal reduction in specific myocardial regions [99]. This variability challenges the traditional view that SERCA2a downregulation is a universal hallmark of HF and reflects the heterogeneous pathophysiology of HFpEF.

SERCA2a is involved in the regulation of intracellular Ca^2+^ homeostasis in cardiomyocytes to maintain normal myocardial contraction-diastole through multiple mechanisms. During systole, cardiomyocytes release large amounts of Ca^2+^ from the sarcoplasmic reticulum in a short period of time through the Ca^2+^ induced-Ca^2+^-release (CICR) mechanism. During diastole, the SERCA2a pump in cardiomyocytes actively transports a large amount of Ca^2+^ to the sarcoplasmic reticulum, thereby shortening the myocardial systole caused by the binding of Ca^2+^ to troponin C [100]. In recent studies, impaired SR Ca^2+^ uptake due to decreased expression and activity of SERCA2a has been found to be one of the important hallmarks of HF [100].

Kho and colleagues [22] showed that protein modification of SERCA2a by small ubiquitin-associated modifiers (SUMOs) at lysine 480 and 585 significantly enhanced its ATPase activity and stability, whereas the levels of SUMO1 and sumoylation of SERCA2a were significantly reduced in the failing heart. Restoration of SUMO1 by adenovirus-associated virus-mediated gene therapy maintains SERCA2a protein abundance and significantly improves cardiac function in HF mice. Furthermore, SUMO modification of SERCA2a at lysine residues 480 and 585 to enhance its ATPase activity is considered a potential therapeutic target for heart failure [22].

In addition, previous studies [82] have indicated that SIRT1, a class III histone deacetylase, and the histone acetyltransferase p300 jointly regulate cardiac SERCA2a activity through modulating acetylation and deacetylation at the K492 (lysine 492) site. Acetylation of the K492 site can significantly reduce SERCA2a activity by interfering with the binding of ATP to SERCA2a. Pharmacological activation of SIRT1, on the other hand, restores SERCA2a activity through deacetylation of the K492 site. These findings may provide a new strategy for the treatment of heart failure.

Other studies have shown that the phosphorylation status of SERCA2 also has an important effect on its activity, for example, specific inhibition of SERCA2 phosphorylation at residue S663 increased SERCA2 pump activity in several human cell types. Fabrice and colleagues [26] demonstrated that the phosphorylation level of SERCA2 at serine 663 serves as a critical regulator of SERCA2 activity and intracellular Ca^2+^ homeostasis. Specifically, this phosphorylation event was found to be elevated in ischemic cardiac tissues from both heart failure patients and mouse models. Mechanistically, blocking phosphorylation at serine 663 significantly enhanced SERCA2 activity and inhibited cell death. This protective effect was mediated by accelerating Ca^2+^ reuptake into the SR/ER, which in turn counteracted Ca^2+^ overload in the cell membrane and mitochondria, and ultimately reduced myocardial infarct size in vivo. In addition, PLN, a negative regulator of SERCA2a, exhibits an inhibitory effect on SERCA2a activity in the nonphosphorylated state [27]. Erwin [101] and Manami [102] have shown experimentally that in model mice that inhibit the phosphorylation of PLN or ablate the PLN gene, the SERCA2a activity was de-repressed and exhibited enhanced contractility and diastolic properties in failing cardiomyocytes, thereby reducing HF mortality.

In addition, histone modifications play a role in the regulation of SERCA2a expression. Numerous studies have also shown that histone modifications can regulate the expression of SERCA2a at the level of *ATP2A2* gene expression, for example, increased expression of histone deacetylases (HDACs) HDAC1 in the heart reduces the level of histone acetylation and thus the expression of SERCA2a. Tian and colleagues [103] found that the reduced acetylation level of histone H3K9 in the *ATP2A2* promoter region of heart failure mice may be related to the increased expression of HDAC1 mRNA, which leads to the expression of the transcription factors GATA4 and Mef2c in the heart. The reduced binding of GATA4 and Mef2c to the *ATP2A2* promoter region leads to the low expression of SERCA2a, which suggests that the acetylation of histone H3K9 in *ATP2A2* promoter region may be related to the reduced expression of SERCA2a in heart failure mice. acetylation of H3K9 may be involved in the regulation of SERCA2a expression.

### 4.3. SERCA2 and Myocardial Hypertrophy

Pathological myocardial hypertrophy is a loss-of-compensation response to increased hemodynamic stress load, leading to myocardial ischemia and reduced myocardial contractility, which further leads to a variety of cardiovascular diseases such as myocardial infarction and heart failure. Myocardial hypertrophy is considered to be a predictor of cardiovascular disease morbidity and mortality, and the study of its pathogenesis and prevention is a hot research topic in the cardiovascular field [104]. Increasing evidence supports the role of Ca^2+^ signaling in the pathogenesis of cardiac hypertrophy.

For example, transgenic (TG)1 mice overexpressing the Ca^2+^-binding protein calmodulin 3-5-fold develop severe cardiac hypertrophy [105]. The CaMKII/SERCA2a pathway is critical in Ca^2+^ signaling in cardiomyocytes. Ca^2+^/calmodulin-dependent protein kinase II (CaMKII) phosphorylates PLN at the Thr17 site, thereby promoting PLN oligomerization, separation from SERCA2a, and de-inhibition of SERCA2a, thereby enhancing the reuptake of calcium ions from the cytoplasm by the sarcoplasmic reticulum [106]. ZHANG and colleagues [107] found that overexpression of CaMKII in mice induced cardiomyocyte hypertrophy, while inhibition of CaMKII overexpression not only inhibited cardiomyocyte hypertrophy, but also improved cardiomyocyte contraction. In addition, Fu and colleagues [108] showed that ubiquitin-specific peptidase 2 (USP2) expression was down-regulated in animal and cellular models of cardiac hypertrophy induced by angiotensin II (Ang II). Moreover, USP2 overexpression inhibited cardiac hypertrophy, calcium overload, and mitochondrial dysfunction in vitro and in vivo. Ang II induced an increase in Ca^2+^ content and expression of calcium overload-associated proteins, t-CaMKII and p-CaMKII, and a decrease in SERCA2 expression, whereas USP2 overexpression attenuated calcium overload. In addition, a related study reported that cardiac function could be restored by gene therapy overexpression of SERCA2 in cardiomyopathy patients with reduced expression of other calcium pumps [109]. Therefore, it is reasonable to speculate whether USP2 overexpression attenuates calcium overload and restores cardiac function in patients with cardiac hypertrophy by upregulating SERCA2.

It has been shown that decreased SERCA function leading to altered calcium ion handling in cardiomyocytes is one of the most striking features of cardiomyocytes in cardiac hypertrophy, and decreasing the activity of β-adrenergic receptors, overexpressing SERCA2 or increasing the Ca^2+^-binding protein S100A1, eliminating the inhibitory effect of PLN on SERCA2a and restoring Ca^2+^ storage in the sarcoplasmic reticulum can be used for the treatment of cardiac hypertrophic diseases [110]. Most and colleagues were the first to demonstrate that myocardial-targeted transgenic overexpression of S100A1 significantly improves cardiac function in mice both under baseline conditions and in response to β-adrenergic receptor (β-AR) stimulation [111]. Cardiomyocyte SR Ca^2+^ cycling is also enhanced after transgenic cardiac S100A1 overexpression, contributing to increased in vivo myocardial contractility. Further, in the current study, they have revealed the novel finding that S100A1 can associate with the cardiac RyR2 isoform and that this interaction may take part in its inotropic mechanism by enhancing SR Ca^2+^ release.

### 4.4. SERCA2 and Atherosclerosis

Atherosclerosis, as the main pathological basis of cardiovascular and cerebrovascular diseases with high morbidity and mortality, such as coronary heart disease and stroke, has a complex pathogenesis. Therefore, it is particularly important to clarify the development mechanism of atherosclerosis to prevent and treat atherosclerosis. Numerous studies have shown that irreversible oxidation of C674 in SERCA2 is present in plaques of both atherosclerotic patients and animal models of atherosclerosis. However, whether the irreversible oxidation of C674 in SERCA2 is directly involved in the process of atherosclerosis and the related regulatory mechanisms need to be elucidated. Tong and colleagues [112] found that the deposition of atherosclerotic plaques in the aorta and aortic valve, the proportion of necrotic nuclei, and macrophage cells in the aortic plaque of SERCA2 C674S mutant knock-in (SKI) mice were significantly lower than those in the aorta. Proportion of necrotic nuclei and macrophage infiltration were significantly increased. In macrophages and endothelial cells, C674 irreversible oxidation increased cytoplasmic Ca^2+^ concentration, increased expression of endoplasmic reticulum stress and inflammatory response marker proteins, and facilitated macrophage-endothelial cell interactions; demonstrating that C674 irreversible oxidation in SERCA2 exacerbates atherosclerosis by inducing endoplasmic reticulum stress to promote inflammatory responses. Another study showed that AMPK-activated protein kinase (AMPKα) is an endogenous inhibitory protein for the oxidation of endothelial sarcoplasmic reticulum/endoplasmic reticulum calcium ATPase [113], and that the expression of endothelial endoplasmic reticulum stress markers was increased in AMPKα-/- LDLR-/- mice, compared with LDLR-/- mice [114], and atherosclerosis was exacerbated. Whether C674 irreversible oxidation in SERCA2 exacerbates atherosclerosis by regulating AMPKα expression remains to be explored. Tong et al. [114] generated a SERCA2 C674S knock-in (SKI) mouse model, in which the cysteine at position 674 of SERCA2 was substituted with serine (S674) to mimic the irreversible oxidative activity loss of C674 under pathological conditions. Their investigations demonstrated that SERCA2 dysfunction results in intracellular Ca^2+^ accumulation and activates Ca^2+^-dependent signaling pathways, such as the calcineurin-mediated nuclear factor of activated T-cells (NFAT) and nuclear factor κB (NFκB) pathways [95], while also downregulating peroxisome proliferator-activated receptor γ (PPARγ) [115] and inducing endoplasmic reticulum (ER) stress [116]. Mechanistically, SERCA2 dysfunction accelerates aortic aneurysm and atherosclerosis by triggering oxidative stress in arterial smooth muscle cells (ASMCs), and concurrently facilitates ASMC phenotypic transformation via the activation of ERK1/2 and angiotensin II/AT1R signaling [114]. Notably, inhibition of oxidative stress in ASMCs alleviates angiotensin II-induced aortic aneurysm and atherosclerosis caused by SERCA2 dysfunction, thereby providing novel insights and potential therapeutic targets for the management of atherosclerosis.

### 4.5. SERCA2 and Diabetic Cardiomyopathy

Diabetes mellitus is a widespread chronic progressive disease, and people with diabetes face a higher risk of coronary artery disease [117], and cardiovascular disease is the leading cause of death in people with diabetes [118]. Diabetic cardiomyopathy (DC) is a diabetes mellitus (DM)-induced pathophysiological condition that can result in HF [119]. Therefore, a comprehensive elucidation of the pathogenesis of CVD and DM is essential for early disease prediction, identification of high-risk populations, and improvement of therapies. Recent studies have shown that hyperglycemia can ultimately cause cardiac pathological changes making them myocardial hypertrophy, fibrosis, cardiac autonomic neuropathy and alterations in cardiac function [28]. And based on these pathological changes, SERCA2a downregulation was found to exist in diabetic cardiac tissues. However, the molecular mechanism of this downregulation is unclear. In addition, it has been demonstrated that hyperglycemia can cause myocardial dysfunction through mechanisms such as direct glucose toxicity, interference with cellular energy metabolism, and imbalance of intracellular Ca^2+^ homeostasis [45]. It is currently believed that oxidative stress-induced polyol activation, glycated proteins can lead to the formation of advanced glycosylation end products (AGEs) and may ultimately contribute to diabetic complications by modulating SERCA2a activity [28].

For example, Bidasee and colleagues [64] found that AGEs were formed on SERCA2a in chronic diabetic myocardial tissues, resulting in a decrease in SERCA2a activity, they suggested for the first time that diabetes could cause downregulation of SERCA2a expression. In their study, they observed that heart tissues from streptozotocin-induced diabetic rats (8 weeks) showed lower levels of SERCA2a and higher levels of PLN, and AGEs were found to cross-link with SERCA2a. In addition, hyperglycemia leads to increased production of ROS and activation of ADP-ribose polymerase 1, an enzyme that inhibits glyceraldehyde 3-phosphate dehydrogenase, which leads to the accumulation of glycolytic intermediates and facilitates the production of AGEs, which in turn glycosylates SERCA2a. This study also showed that diabetic rats (6 weeks) starting treatment with insulin for 2 weeks not only significantly improved cardiac function, but also prevented the formation of cross-links of AGEs on SERCA2a. In addition to the reduced expression of SERCA2a, the increase in PLNs and the decrease in the SERCA2a-PLN ratio, and the prolongation of the cardiac diastolic interval may also be due to the formation of AGEs [64]. The Dillmann group [120] systematically investigated the temporal changes in the expression levels of SERCA2a and PLN in a mouse model of type 2 diabetes mellitus (DM2). At 4 months after DM2 induction, SERCA2a protein levels were significantly decreased, and this reduction was associated with a lowered ratio of phosphorylated PLN (p-PLN) to total PLN (p-PLN/PLN). Additionally, PLN is subject to O-linked N-acetylglucosamine (O-GlcNAc) modification, and the level of PLN O-GlcNAcylation was markedly elevated in DM2 mice. The study further revealed an inverse correlation between p-PLN levels and O-GlcNAcylated PLN levels in DM2 mice. O-GlcNAcylation was shown to interfere with the phosphorylation of PLN, leading to the accumulation of non-phosphorylated PLN, which in turn exacerbates systolic dysfunction [121].

In addition, it has been shown that the polyol pathway may also impair SERCA2a activity by increasing oxidative stress [122]. This metabolic pathway is not active at normal blood glucose levels because aldose reductase (AR), the rate-limiting enzyme of the polyol pathway, has a very high K_m_ for glucose. However, at high blood glucose levels, AR reduces glucose to sorbitol while its cofactor NADPH is oxidized to form NADP. Sorbitol is then converted to fructose by sorbitol dehydrogenase (SDH), accompanied by the reduction of NAD^+^ to NADH [122]. Since NADPH is an important coenzyme for the regeneration of glutathione reductase to GSH, its reduced level further leads to lower levels of reduced glutathione (GSH). On the other hand, elevated NADH levels also increase intracellular superoxide [123]. Thus, at high blood glucose levels, hyperglycemia not only reduces antioxidant capacity by activating the polyol pathway, but also by increasing ROS levels, ultimately leading to oxidative stress. Previous findings showed that cardiac AR activity was elevated in diabetic mice and AR inhibitor treatment improved contractility of papillary muscles in diabetic rats, but the mechanism is not clear. Tang and colleagues [124] found that the polyol pathway resulted in acute high glucose-induced cardiac contractile dysfunction by decreasing the activity of SERCA2. In the acute hyperglycemic heart, activation of the polyol pathway increased the NADH/NAD+ ratio as SDH oxidized sorbitol to fructose. The increased NADH stimulates NADH oxidase to produce ROS. ROS inhibit SERCA2a by oxidizing cysteine thiols, interfering with ATP-binding sites and rendering them incapable of hydrolyzing ATP [125]. Tang and colleagues also demonstrated that hyperglycemic perfusion resulted in increased nitration of SERCA2a, elevated levels of SERCAC674-SO3H, and that, in addition, the addition of AR inhibitor or SDH inhibitor added to high glucose perfusate decreased SERC2a nitrification and SERCAC674-SO3H levels, and these evidences suggest that the decrease in SERCA2a activity is due to high glucose-induced oxidative stress. Taken together, the polyol pathway is the main cause of high glucose-induced oxidative stress.

A recent study showed that administration of insulin to type 2 diabetic rats resulted in an early increase in the SERCA2a/PLN ratio and an increase in the diastolic rate of cardiomyocytes, suggesting that insulin directly stimulates SERCA2a expression in cardiomyocytes [124]. This means that insulin and SERCA2a play an important role in the systolic-diastolic process of type 2 diabetic heart. This study also found that insulin induced Akt (v-Akt Murine Thymoma Viral Oncogene) phosphorylation [126], suggesting that the elevated SERCA2a levels after administration of insulin therapy may result from the PI3-kinase-Akt-SERCA2a signaling pathway [127]. Interestingly, endoplasmic reticulum stress also plays an important role. Takada and colleagues used the OLETF (spontaneous type 2 diabetes) rat model, cultured for 25–30 weeks, and found that levels of endoplasmic reticulum stress markers CRP78 and GRP94 were increased in cardiomyocytes, whereas the SERCA2a protein was decreased (35%) [128]. Further studies found elevated levels of SERCA2a ubiquitination compared to nondiabetic rats (LETO), demonstrating that endoplasmic reticulum stress mediates enhanced SERCA2a ubiquitination, resulting in down-regulation of SERCA2a protein expression, which in turn induces diastolic insufficiency in the heart. However, numerous studies have shown that SERCA2a mRNA levels are reduced in diabetic rats, whereas the above study model does not. A plausible explanation for the findings is that the reduction in SERCA2a protein may be preceded by some nontranscriptional mechanisms, such as posttranslational modifications, that reduce SERCA2a, and that the reduction in SERCA2a mRNA as a result of SERCA2a gene transcription is a late event in diabetes mellitus. In conclusion, in type 2 diabetes, insulin may regulate SERCA2a protein expression through direct stimulation of SERCA2a, whereas insulin resistance can induce endoplasmic reticulum stress.

In summary, SERCA2 plays an important role in a variety of cardiovascular diseases, and its dysfunction is closely related to the occurrence and development of cardiovascular diseases. An in-depth study of the regulatory mechanism of SERCA2 is expected to provide new targets and strategies for the prevention and treatment of cardiovascular diseases (Table 2).

## 5. Advances in Treatment Strategies

In recent years, significant progress has been made in therapeutic strategies for cardiovascular disease targeting SERCA2 dysfunction, mainly in the direction of small-molecule activators, gene therapy, indirect regulatory strategies, and drug repositioning. These interventions aim to restore intracellular Ca^2+^ homeostasis, improve myocardial systolic and diastolic function, and slow the progression of cardiovascular disease.

### 5.1. Small Molecule Activator

Small-molecule activators of SERCA2 improve Ca^2+^ uptake efficiency by directly enhancing ATPase activity or protein stability. Among them, the quinolinamide compound CDN1163, the first SERCA2 variant activator, has demonstrated significant efficacy in animal models of heart failure [129]. CDN1163 enhances SERCA2a activity, reduces sarcoplasmic reticulum Ca^2+^ leakage, and improves myocardial contractile function [130]. Kang and colleagues [131] demonstrated that in an animal model of insulin resistance and type 2 diabetes mellitus, activation of the SERCA2b isoform by CDN1163 in the liver reduced ER stress and increased mitochondrial efficiency and metabolic parameters, suggesting that SERCA activators may be promising agents for the treatment of diabetes and metabolic dysfunction. Additionally, novel compounds such as NSC191803 and CDN1163 derivatives have shown potential in preclinical studies; however, their selectivity and pharmacokinetic properties need further optimization [132].

### 5.2. Gene Therapy

Gene therapy aims to compensate for the lack of functional SERCA2a expression by delivering the SERCA2a gene. Greenberg et al. [133] found in a phase 2b pilot study of intracoronary administration of AAV1/SERCA2a in patients with advanced heart failure improved intracellular Ca^2+^ levels by increasing the SERCA2a protein levels, thereby restoring diastolic and systolic function. Adenoviral (AV) and adeno-associated viral (AAV) vectors are the most commonly used carriers in cardiovascular gene therapy. Although AV vectors have achieved some success in transducing SERCA2a for heart failure therapy, their transgene expression is transient and highly immunogenic. Consequently, AAV vectors are predominantly used due to their low immunogenicity and high transfection efficiency, demonstrating significant efficacy in animal models targeting SERCA2a [27]. AAV9-SERCA2a (adeno-associated viral serotype 9 vector) showed safety in early clinical trials (e.g., the CUPID 1/2 trial) but failed to significantly improve the prognosis of heart failure patients in phase III studies [134]. Reasons for this failure may include: (1) Insufficient efficiency of viral delivery: Low myocardial transfection rate; (2) Immunogenicity problems: Some patients produce AAV9-neutralizing antibodies; (3) Disease stage dependence: Myocardial fibrosis affects efficacy in patients with advanced heart failure. Optimized vectors (e.g., engineered AAV variants) and combination therapies (e.g., antifibrotic drugs) are currently being explored [135].

Jiang et al. [136] established a homozygous *NEXN*-knockout cardiomyocyte model by combining CRISPR/Cas9 gene-editing technology with the directed differentiation of human induced pluripotent stem cells (hiPSCs). Their findings demonstrated that *NEXN*^−^/^−^ hiPSC-CMs exhibited a hypertrophic phenotype, accompanied by a marked reduction in SERCA2a expression and impaired Ca^2+^ handling in *NEXN*^−^/^−^ hiPSCs. Notably, treatment with the metabolism-enhancing agent levocarnitine and SERCA2a Activator 1 was shown to effectively ameliorate the energy metabolism of *NEXN*^−^/^−^ hiPSCs. In a separate study, Zhang et al. [137] reported that overexpression of the long non-coding RNA (lncRNA) ZFAS1 in otherwise healthy mice induced cardiac dysfunction analogous to that observed in a murine model of myocardial infarction. At the molecular level, they revealed that ZFAS1 directly binds to the SERCA2a protein, thereby suppressing both its activity and expression. Critically, knockdown of ZFAS1 reversed the inhibitory effect of this lncRNA on SERCA2a function. Unlike lncRNAs, Pan et al. [138] performed transcriptome analysis following circRYR2 knockdown and found that this intervention led to decreased levels of SERCA2 and calsequestrin (CSQ), as well as altered phosphorylation of PLN. However, overexpression of circRYR2 in hiPSC-CMs did not affect the total protein abundance of SERCA2, indicating that circRYR2 modulates SERCA2 activity through mechanisms independent of altering its synthesis or degradation rates. Collectively, these observations highlight the need for further investigations into the specific molecular mechanisms underlying the interactions between circRYR2, SERCA2, and its regulatory factors (PLN and NCX1) under both physiological and pathological conditions.

### 5.3. Indirect Regulation Strategies

Since SERCA2 is regulated by multiple endogenous factors, indirect intervention strategies are promising:

(1) Phosphoreceptor natriuretic protein inhibition: PLN is a major negative regulator of SERCA2. Small molecules targeting PLN (e.g., S68A mutant mimetic peptide) or gene silencing can enhance SERCA2 activity [85]. Previous studies have shown that type 1 protein phosphatase (PP1a) is the major phosphatase responsible for PLN dephosphorylation [139]. Wu et al. [140] further demonstrated that pretreatment with lignans (3′,4′,5′,7′-tetrahydroxyflavonoids) in adult rat cardiomyocytes decreased PLN protein levels by reducing PP1a, which resulted in an increase in SERCA2a expression. In addition, Li et al. [141] found that MiR-221 could directly bind to the 3′UTR of PLN mRNA to target PLN. Restoration or up-regulation of MiR-221 levels after myocardial ischemia–reperfusion injury (MIRI) may be a novel therapeutic strategy to attenuate the deleterious effects of PLN up-regulation and the resulting calcium overload.
(2) Phosphoprotein Inhibition: PLN is a key regulator of Ca^2+^ homeostasis and contractility in the heart. Targeting SERCA2a/PLN activity restores cardiac contractile function and benefits cardiac diastole [29]. PLN forms a complex with SERCA2a and inhibits its function, whereas phosphorylation of PLN during β-adrenergic stimulation rescinds its inhibitory effect [85]. Recent evidence suggests that heat shock-associated protein X-1 (HAX-1) has been found to interact directly with PLN [142] and enhances the inhibitory effect of PLN on SERCA2a by stabilizing the dephosphorylation of PLN, thereby reducing the contractility of cardiomyocytes [143]. In addition, chemically synthesized drugs such as istaroxime have been found to interact directly with the PLN/SERCA2a complex, leading to dissociation of PLN from SERCA2a, thereby accelerating Ca^2+^ cycling [144]. Shah et al. investigated the effect of istaroxime on diastolic stiffness in patients with acute HF, and their findings showed that Istaroxime decreased pulmonary capillary wedge pressure (PCWP), increased systolic blood pressure (SBP) and reduced diastolic stiffness in patients with acute heart failure syndrome [145]. Torre et al. [146] found that istaroxime increased myocardial diastole in a diabetic rat model by activating SERCA2a and improving Ca^2+^ homeostasis. Meanwhile, Kaneko et al. [147] discovered that pyridone derivatives acted similarly to istaroxime.
(3) MicroRNA regulation: Recent studies have shown that the dysfunction of the 3′- and 5′-UTRs, which are important regulators of mRNA translation, has been associated with the pathophysiology of various diseases [148]. MicroRNAs (MiRNAs, MiR) are negative regulators of mRNA translation, regulating physiological functions and contributing to various diseases by binding to the 3′-UTRs of target mRNAs [149]. Vandecaetsbeek et al. [30] found that conserved regions of the 3′-UTR of SERCA2 may act as post-transcriptional binding sites for MiRNAs. Wahlquist further discovered that MiR-25 is a key repressor of SERCA2a and cardiac function during heart failure. MiR-25 is overexpressed in heart failure and inhibits SERCA2a translation, and its antisense oligonucleotide (antagoMiR-25) restores cardiac function in animal models [49]. Anti-MiR-25 injection restored SERCA2a protein expression as well as post-translational modifications of SERCA2a, including a significant increase in sumoylated SERCA2a, which enhances transporter protein stability and ATPase activity [22]. Meanwhile, Lei et al. [150] found that MiR-132/212 could inhibit SERCA2a expression by binding to the SERCA2a 3′-untranslated region (3′-UTR), impairing the contractility of cardiomyocytes in failing hearts. This effect can be reversed by using microRNA inhibitors antiMiR-132 or antiMiR-212 (AMO), respectively. Furthermore, Williams et al. [151] discovered that hypoxia-inducible factor (HIF)-1-dependent up-regulation of MiR-29c inhibited SERCA2 expression in a transgenic overexpression system, reducing cardiomyocyte contractility. This inhibition was ameliorated by administration of a MiR-29c antagonist sequence (antimir).
(4) Redox regulation: Oxidation of Cys674 in SERCA2 can lead to inactivation, and antioxidants (e.g., thioredoxin mimics) may provide protection [152]. Que et al. demonstrated that the redox state of C674 in SERCA2 is critical for maintaining the balance between the calmodulin phosphatase-mediated NFAT/NF-κB pathway and PPARγ. Irreversible oxidation of C674 promotes aortic aneurysm development by inducing SMC phenotypic modulation through an imbalance between enhanced activation of the calmodulin phosphatase-mediated NFAT/NF-κB pathway and decreased PPARγ expression [115].

### 5.4. Drug Repositioning

Certain drugs have been found to unexpectedly modulate SERCA2 activity: (1) Sildenafil (PDE5 inhibitor): In addition to its classical vasodilatory effects, sildenafil enhances SERCA2 expression via the NO/cGMP pathway and improves Ca^2+^ handling in a diabetic cardiomyopathy model [153]. (2) Digoxin (Na^+^/K^+^-ATPase inhibitor): At low doses, digoxin may indirectly affect SERCA2 function through calcium sensitization; however, caution is needed due to potential toxicity [154]. (3) Statins: In addition to their lipid-lowering effects, simvastatin may upregulate SERCA2 expression via the RhoA/ROCK pathway [155].

## 6. Summary

Cardiovascular disease (CVD) presents a complex and diverse challenge to global health. In recent years, increasing attention has been directed toward the pivotal role of dynamic intracellular calcium ion (Ca^2+^) fluctuations in the pathogenesis of CVD. Dysfunction of SERCA2, a key enzyme regulating intracellular Ca^2+^ concentrations, has been identified as a critical factor in the development of numerous cardiovascular disorders. SERCA2 is mainly responsible for pumping intracellular calcium ions back to the sarcoplasmic reticulum/endoplasmic reticulum, thus maintaining the homeostasis of intracellular calcium ions. The expression of SERCA2 is regulated by various factors at multiple levels.

At the transcriptional level, the transcription factors such as TFAM, TFB2M, and SP1 bind to the *ATP2A2* promoter region to regulate SERCA2a transcriptional activity. In addition, the acetylation of histone H3K9 in the *ATP2A2* promoter region influences SERCA2a expression. The acetyltransferase p300 can interfere with ATP binding to SERCA2a by acetylating the K492 site, thereby reducing its activity. Conversely, SIRT1 can restore SERCA2a activity through deacetylation of the K492 site. At the molecular level, the amino acid modifications of SERCA2 at specific sites are closely linked to its activity. For example, inhibition of phosphorylation at serine 663 significantly enhances SERCA2 activity, preventing cell death by counteracting mitochondrial Ca^2+^ overload through increased Ca^2+^ reuptake. Furthermore, irreversible oxidation of cysteine 674 (C674) in SERCA2 elevates cytoplasmic Ca^2+^ levels in macrophages and endothelial cells, exacerbating atherosclerosis by inducing endoplasmic reticulum stress and promoting an inflammatory response. Interestingly, in pulmonary artery smooth muscle cells, irreversible oxidation of C674 by ROS does not directly alter SERCA2 mRNA or protein expression levels. Instead, it upregulates cell cycle-related proteins through activation of the IRE1α-XBP1s pathway, accelerating the cell cycle and promoting cellular proliferation.

Recent studies highlight the complexity and diversity of the Ca^2+^ signaling network, with SERCA2 serving as a crucial hub in various cardiovascular diseases. For instance, SERCA2a is a key determinant of cardiac systolic and diastolic regulation. Reduced expression and activity of SERCA2a can lead to cardiac systolic and diastolic dysfunction, contributing to the progression of conditions such as pulmonary hypertension, atherosclerosis, and diabetic cardiomyopathy. SERCA2b, often referred to as a “housekeeping gene,” maintains basal Ca^2+^ homeostasis across various cell types, while SERCA2c may function in a paracrine capacity in certain cells. Additionally, there is a notable association between SERCA2 dysfunction and diabetic bone damage. In type 2 diabetes (T2D), reduced expression and activity of SERCA2 disrupt the balance between SERCA-mediated Ca^2+^ uptake and Ca^2+^ release from IP3 receptors and ryanodine receptors, as reported by reference. Treatment with SERCA2 agonists can restore Ca^2+^ dynamics in osteocytes, thereby ameliorating bone injury associated with T2D.

Despite significant advancements in understanding SERCA2’s role in the mechanisms underlying cardiovascular and age-related oxidative diseases, as well as its implications in clinical diagnosis and treatment, numerous scientific questions remain unanswered. Key questions include: What are the specific regulatory mechanisms governing SERCA2 expression? What are the distinct isoforms of SERCA2, and how do their mechanisms of action differ in cardiomyocytes and vascular smooth muscle cells? Is there a correlation between the various targets that regulate SERCA2 activity? How can SERCA2-related regulatory mechanisms be leveraged to develop biologics targeting SERCA2, and what are the interactions between SERCA2 and other Ca^2+^ transporters?

Future research could focus on the following key areas: (1) In-depth study of SERCA2 regulators and their mechanisms of action: Investigate the various regulators of SERCA2 and elucidate their mechanisms of action to facilitate the development of targeted therapeutic agents. Understanding how these regulators interact with and modulate SERCA2 activity is crucial for designing effective interventions. (2) Exploring the interactions between SERCA2 and other calcium regulator proteins (e.g., PLN, RyR, etc.): Examine the interactions between SERCA2 and other calcium regulatory proteins, such as PLN and ryanodine receptors (RyR), to achieve a more comprehensive understanding of cardiac calcium signaling. This holistic perspective will enhance our knowledge of calcium homeostasis and its impact on cardiac function. (3) Utilizing of gene editing and stem cell technology for gene therapy application: Employ gene editing technologies (e.g., CRISPR/Cas9) and stem cell technologies to explore the potential of gene therapy targeting SERCA2 in the treatment of cardiovascular diseases. These advanced techniques offer promising avenues for correcting SERCA2 dysfunction and improving clinical outcomes. By addressing these areas, future studies will advance our understanding of SERCA2’s role in cardiovascular physiology and pathology, paving the way for innovative diagnostic and therapeutic strategies.

**Figure 4:**
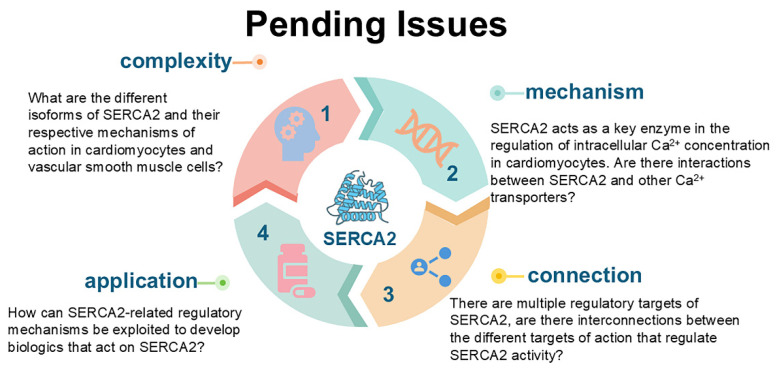
A diagram of several key scientific issues that remain to be addressed in the future.

In conclusion, as our understanding of the relationship between SERCA2 and cardiovascular diseases deepens and research methodologies, as well as gene diagnosis and treatment technologies, become more advanced, investigations into Ca^2+^ regulatory proteins centered around SERCA2 are poised to uncover new pathways for understanding the mechanisms underlying CVDs. This progress is expected to enhance clinical diagnosis, treatment strategies, and prognostic assessments for cardiovascular diseases (Figure 4).

## Figures and Tables

**Table 1:** Comparison Table of Expression Patterns, Affinity and CVD Impact of SERCA2 Subtypes.

Subtype	Main Expression Organization	Ca^2+^ Affinity	Association with CVD	Reference
SERCA2a	Cardiac muscle, skeletal muscle, and vascular smooth muscle (present in small amounts)	Medium	Down-regulation of this expression can lead to dysfunction of myocardial contraction and relaxation, causing heart failure; it is also involved in vascular remodeling in hypertension.	[32]
SERCA2b	Vascular endothelial cells, vascular smooth muscle cells	High	Functional defects can promote inflammatory responses in endothelial cells, accelerate atherosclerosis; and induce vascular remodeling in pulmonary arterial hypertension.	[33]
SERCA2c	Extensive organization (low expression)	Low	The upregulation of expression may alleviate the calcium overload that occurs after vascular damage, but the specific mechanism remains unclear.	[34]

**Table 2:** Studies on the mechanisms of SERCA2 in cardiovascular disease.

Disease	Pathogenesis	SERCA2 Activity Changes	Reference
Pulmonary hypertension	Nox4 promotes irreversible oxidation of SERCA2 C674 by generating ROS, which in turn exacerbates vascular injury inducing promotion of pulmonary vascular remodeling and development of PH	deactivation	[94]
Heart failure	SERCA2a protein modification by small ubiquitin-associated modifiers at the K480 and K585 sites significantly enhances enzyme activity and stability	upregulation	[22]
	SIRT1 and p300 Significantly Reduce SERCA2a Activity by Regulating K492 Site Acetylation	downregulation	[82]
	Phosphorylation of SERCA2 at serine 663 is elevated in ischemic hearts of HF patients and mice, and blocking serine 663 phosphorylation significantly increases SERCA2 activity	downregulation	[26]
Myocardial hypertrophy	CaMKII phosphorylates PLN at the Thr17 site to promote PLN oligomerization, separation from SERCA2a, and de-inhibition of SERCA2a, thereby enhancing the ability of the sarcoplasmic reticulum to re-uptake calcium from the cytosol	upregulation	[106]
Atherosclerosis	C674 Irreversible oxidation in SERCA2 Exacerbates Atherosclerosis by Inducing Endoplasmic Reticulum Stress to Promote Inflammatory Responses	downregulation	[112]
Diabetic cardiomyopathy	AGEs form on SERCA2a in diabetic myocardial tissues, resulting in reduced SERCA2a activity	downregulation	[64]
	Polyol Pathway Impairs SERCA2a Activity by Increasing Oxidative Stress at High Blood Glucose Levels	downregulation	[122]

## Data Availability

No new data were created or analyzed in this study.

## Abbreviations

The following abbreviations are used in this manuscript:

CVDs	Cardiovascular diseases
SERCA2	Sarcoplasmic/endoplasmic reticulum Ca^2+^-ATPase 2
Ca^2+^	Calcium
WHO	World Health Organization
TM	Transmembrane
PLN	Phospholamban
HDAC	Histone deacetylase
TFAM	Mitochondrial transcription factor A
TFB2M	Mitochondrial transcription factor B2
PTMs	Post-translational modifications
ROS	Reactive oxygen species
DCM	Dilated cardiomyopathy
Cys674	S-glutathionylation of cysteine 674
SUMOs	Small ubiquitin-associated modifiers
shRNA	Small hairpin RNA
NATs	N-terminal α-aminoacetylation
KATs	Lysine ε-aminoacetylation
KDACs	KATs and lysine deacetylases
K492	Lysine 492
SPEG	Rhabdomyosin-specific protein kinase
MLCK	Troponin light chain kinase
SLN	Sarcolipin
PH	Pulmonary hypertension
PASMC	Pulmonary artery smooth muscle cells
Nox4	NADPH Oxidase 4
CDK	Cyclin-dependent kinase
HF	Heart failure
CICR	Ca^2+^ induced-Ca^2+^-release
CaMKII	Calcium/calmodulin-dependent protein kinase II
USP2	Ubiquitin-specific peptidase 2
Ang II	Angiotensin II
AMPKα	AMPK-activated protein kinase
AGEs	Advanced glycosylation end products
AR	Aldose reductase
SDH	Sorbitol dehydrogenase
GSH	Glutathione
ATP	Adenosine triphosphate
Akt	V-Akt Murine Thymoma Viral Oncogene
OLETF	Spontaneous type 2 diabetes
AV	Adenoviral
AAV	Adeno-associated viral
MIRI	Myocardial ischemia–reperfusion injury
HAX-1	Heat shock-associated protein X-1
PCWP	Pulmonary capillary wedge pressure
SBP	Systolic blood pressure
T2D	Type 2 diabetes
HIF	Hypoxia-inducible factor
